# Association Between Socio-Affective Symptoms and Glutathione and CD4 and CD8 Lymphocytes in College Students

**DOI:** 10.3389/fpsyg.2021.666347

**Published:** 2022-01-05

**Authors:** Cecilia Luz Balderas-Vazquez, Blandina Bernal-Morales, Eliud Alfredo Garcia-Montalvo, Libia Vega, Emma Virginia Herrera-Huerta, Juan Francisco Rodríguez-Landa, José Felipe Velázquez-Hernández, María del Carmen Xotlanihua-Gervacio, Olga Lidia Valenzuela

**Affiliations:** ^1^Programa de Doctorado en Neuroetología, Instituto de Neuroetología, Universidad Veracruzana, Xalapa, Mexico; ^2^Laboratorio de Neurofarmacología, Instituto de Neuroetología, Universidad Veracruzana, Xalapa, Mexico; ^3^Facultad de Ciencias Químicas, Universidad Veracruzana, Orizaba, Mexico; ^4^Departamento de Toxicología, Centro de Investigación y de Estudios Avanzados del Instituto Politécnico Nacional, Ciudad de México, Mexico; ^5^Programa de Doctorado en Ciencias Biomédicas, Centro de Investigaciones Biomédicas, Universidad Veracruzana, Xalapa, Mexico

**Keywords:** stress, glutathione, lymphocyte, depression symptoms, anxiety symptoms, college students

## Abstract

**Background:** The prevalence of anxiety and depression in young students is associated with biosocial factors and scholastic stress. However, few studies have evaluated emotional-affective symptoms that are related to the immune system and antioxidant parameters in young individuals without diagnoses of affective disorders.

**Aim:** This study aims to assess the relationship between emotional-affective symptoms and glutathione concentrations and CD4 and CD8 lymphocyte counts in college students.

**Methods:** College students (*n* = 177) completed standardized psychometric instruments, including the Perceived Stress Scale, Hamilton Anxiety Scale, Beck Depression Inventory, Familiar Social and Friends Support Scale, and Rosenberg Scale. Blood samples were biochemically analyzed. Analyses of variance were conducted between four groups according to symptom severity.

**Results:** A considerable prevalence of stress, anxiety, and depression symptoms was observed and negatively correlated with self-esteem and socio-familiar support. Perceived stress was sexually dimorphic. Although biochemical parameters were within reference ranges, glutathione, CD4, and CD8 tended to be lower in participants with anxiety and depression symptoms, which may be of predictive value.

**Conclusion:** The relationship between antioxidant/immune parameters and socio-affective scores is latent in undiagnosed college students who might develop affective disorders. The findings suggest that during the initial development of affective disorders, stress management strategies should be implemented to help college students cope with the academic load and monitor negative changes in their physiological state.

## Introduction

Research on the etiology of stress in college students who are studying health and engineering has gained importance in recent years because they are exposed to higher levels of stress than others (Balaji et al., 2019), affecting as many as 60% of young people (Fasoro et al., 2019; Kumar et al., 2019). Scholastic stress can occur during professional training, generating continual states of uncertainty (Oura et al., 2020). Although a certain level of alertness is necessary to adequately complete scholastic tasks (Luna et al., 2020), excess responsibilities, high workloads, teacher evaluations, competitiveness, a fear of failure, stipulated goals by parents, cognitive fatigue, and alterations of sleep and eating habits can surpass the level of stress that students can handle (Pozos-Radillo et al., 2016; Luna et al., 2020). An adequate level of socio-familiar support and self-esteem helps students cope with adverse situations. Families are the primary social nucleus that provides social, emotional, and economic support for the development of the individual (Yubero et al., 2018). An adequate level of familiar support is correlated with high self-esteem. Both socio-familiar support and self-esteem are determinant predictors of academic achievement and adaptation to changes that are associated with professional education (Yubero et al., 2018; Choi et al., 2019). Thus, poor levels of these factors can worsen the severity of depressive traits (Lamis et al., 2016).

High and chronic stress negatively affects physical health and promotes psychopathological symptoms (Jiménez-Ortiz et al., 2019). The prevalence of anxiety and depression is 20–60% in college students (Dalky and Gharaibeh, 2018; Liu et al., 2019). Comorbidities among anxiety and depression can raise suicide risk (Becker et al., 2018). Moreover, anxiety and depression symptoms negatively affect executive function, motivation, and the ability to learn, lowering the likelihood of success (Bernal-Morales et al., 2015; Moreira de Sousa et al., 2018; Khesht-Masjedi et al., 2019).

Stress is well known to affect bodily functions, such as causing the inadequate neutralization of free radicals (Yaribeygi et al., 2017). Oxidative stress and antioxidant deficits are observed when the hypothalamic-pituitary-adrenal (HPA) axis is excessively activated (Coronado et al., 2015). These changes lower the availability of neurotrophic factors and the availability of monoaminergic neurotransmitters (McEwen, 2017). Thus, oxidative damage in depression and anxiety disorders can affect the amygdala and hippocampus (Salim, 2017), among other brain structures, reduce neurogenesis (Hassan et al., 2014; van Velzen et al., 2017), and contribute to cognitive dysfunction (Duffy et al., 2014). Glutathione (GSH; γ-L-glutamyl-L-cysteinylglycine) is a water-soluble tripeptide component of the cell cytoplasm and an important first-line intracellular antioxidant that protects against oxidative damage (Denzoin Vulcano et al., 2013). The relationship between oxidative stress and inflammatory processes has been demonstrated by an increase in plasma markers in depression (Black et al., 2015; Lindqvist et al., 2017), such as proinflammatory cytokines (Slavich et al., 2020). Additionally, an imbalance and reduction of T cells that derive from glucocorticoid resistance and the accumulation of neurotoxic products that are generated by both the inflammatory process (Haapakoski et al., 2016) and oxidative stress are linked to the presence and severity of depression symptoms (Goyal et al., 2017; Jeon et al., 2018).

Research on the scope of stress in college students has mainly analyzed psychosocial factors, cognitive processing, and personality characteristics of individuals, to detect anxiety or depression symptoms and to a lesser extent the association between stress and the immune system. Based on the importance of identifying biochemical correlates, this study investigated GSH levels, CD4 and CD8 lymphocyte counts, and their associations with perceived stress, anxiety and depression symptoms, self-esteem, and socio-familiar support in young adults without a prior clinical diagnosis. We hypothesized that less perceived support and lower concentrations of these antioxidant and immune parameters underlie symptoms of anxiety and depression in healthy young adults.

## Materials and Methods

### Participants and Procedures

A cross-sectional study was performed on male and female college students (≥18 years old) who were enrolled in the first semester (August–November 2018) of educational programs in chemical sciences.

The sample size of 107 students was calculated based on a 95% confidence interval and 90% statistical power from previous studies that were conducted with Mexican young adults. The sample size was corrected according to the 1,283 students who were enrolled in the Faculty of Chemical Sciences and an expected non-response rate of 30%.

The exclusion criteria included the identification of and previous treatment for psychiatric disorders. Diagnoses of psychiatric or affective disorders were determined by asking the following questions: “Do you have a psychiatric or affective diagnosis?” “Have you taken a pharmacological treatment or received psychological therapy?” Additionally, some medical conditions exceed the normal range of hematological parameter counts and antioxidant levels. Therefore, students with asthma, diabetes mellitus, anemia, autoimmune diseases, cancer, human immunodeficiency virus, and current infection were excluded from the study. These medical conditions were assessed by asking absence/presence questions. The exclusion criteria also included an incomplete questionnaire, the lack of a blood sample, and leukocyte counts or hemoglobin concentrations outside the reference range. Hemoglobin concentrations (sodium lauryl-sulfate reaction) and total leukocyte counts (electrical impedance) were determined using a hematological analyzer (ADVIA 60, Siemens, Munich, Germany).

A total of 201 students were invited as the initial sample. The rate of response was 97.01% (i.e., 195 participants completed the questionnaire, of whom 188 provided blood samples). Eleven students were excluded from the study because their hematological analyses were outside the reference range. The final sample size was 177.

### Ethics Statement

The project was approved by the Research Ethics Committee of the Institute of Health Sciences of the university (registration no. CE-ICS No._002, 2018). The latest Helsinki Code principles and Mexican Regulations on Health Research were followed. Students were invited to participate in the study in the school setting during a talk that provided information on the project and exclusion criteria. The participants provided written informed consent to participate in the study. Participation was voluntary and financial or school incentives were not provided for participation.

### General Characteristics of the Participants

A structured questionnaire was administered to ascertain general aspects [e.g., age, occupation, marital status, residence, substance use, reasons of substance use (e.g., for pleasure, social use, nervousness, or sadness), physical activity], academic aspects, and stressors in school. The menstrual cycle phase was determined for women at the time of the study to avoid possible confounding effects on anxiety and depression symptoms. The menstrual cycle was recorded as regular or irregular and stratified into four phases according to the number of days after beginning menses: menstrual (1–6 days), follicular (7–14 days), luteal (15–24 days), and premenstrual (≥25 days; Favre and Serrador, 2019).

### Psychometric Variables

Stress was assessed using the Perceived Stress Scale (PSS-10; Cohen et al., 1983), which measures the degree to which life situations are seen as stressful during the last month. It has been validated in the population of interest (Cronbach’s α>0.81; González Ramírez and Landero Hernández, 2007). It contains 10 statements with five response options each, rated from 0 to 4 points. The scale provides a stress score as follows: 0–10, no stress; 11–20, mild stress; and 21–40, high stress.

The Hamilton Self Rating Scale for Anxiety assesses the severity of anxiety symptoms (Cronbach’s α = 0.80; Hamilton, 1959). It has been validated in the population of interest (Flores Ocampo et al., 2007). It includes 14 statements, with five possible responses each, rated from 0 to 4 points. The scale provides an anxiety score as follows: 0–6, no anxiety symptoms; 7–12, mild anxiety symptoms; 13–34, moderate anxiety symptoms; and 35–56, severe anxiety symptoms.

The Beck Depression Inventory I (BDI-I) was used to evaluate depression symptoms, which assesses psychic, physical, and cognitive aspects, not including anxiety symptoms (Beck et al., 1996). It has been validated in the population of interest (Cronbach’s α = 0.87; Jurado et al., 1998). It consists of 21 statements with four responses each, rated from 0 to 3 points, according to severity. The scale provides a depression score as follows: <11, absence of depression symptoms, 11–20, mild depression symptoms; and ≥21, moderate depression symptoms.

The Rosenberg Scale was used to assess self-esteem (Rosenberg, 1965). It is a self-assessment of attributes, skills, and abilities. It has been validated in the young population (Cronbach’s α = 0.79; Jurado Cárdenas et al., 2015). To control for the effect of acquiescence, five of 10 statements are written in a negative sense (3, 5, 8, 9, and 10). Responses are recorded on a Likert-type scale, from 4 to 1. Responses on the other five statements are recorded from 1 to 4. The total score is stratified into three levels as follows: <25, low self-esteem; 26–29, average self-esteem; and 30–40, high self-esteem.

The Familiar Social and Friends Support Scale (FSFSS) was used to assess social and familiar support (Procidano and Heller, 1983). It has been validated in the population of interest (Cronbach’s α = 0.79; González Ramírez and Landero Hernández, 2014). It assesses support received in the familiar and social dimensions. It consists of 15 statements with five response options, rated from 1 to 5. Familiar support is evaluated by statements 1, 3, 5, 7, 9, 11, 13, and 14. Social support is evaluated by statements 2, 4, 6, 8, 10, 12, and 15. There are no defined cut-off points. The minimum score is 15, and the maximum score is 75. Higher scores indicate more social and familiar support.

### Groups

The students were stratified into the following four groups to analyze self-esteem, socio-familiar support, GSH levels, and CD4 and CD8 lymphocyte counts according to PSS-10, Hamilton Anxiety Scale, and BDI-I scores: control group (participants without stress, anxiety, or depression), stress (S) group (PSS-10 score ≥11), stress and anxiety (SA) group (PSS-10 score ≥11 and Hamilton Anxiety Scale score ≥7), and stress, anxiety, and depression (SAD) group (PSS-10 score ≥11, Hamilton Anxiety Scale score ≥7, and BDI-I score ≥11). We generated four groups because the phenomenology of stress, anxiety, and depression share symptoms (Ramón-Arbués et al., 2020) and are also a reference for possible comorbidities, which are common.

### Blood Testing

Blood samples were collected by arm venipuncture 3–7 days after the students completed the questionnaire and scales. Phlebotomy was performed between 7:00 and 8:30 a.m. using the anticoagulant ethylenediaminetetraacetic acid (EDTA; Vacutainer, Becton Dickinson Biosciences, Franklin Lakes, NJ, United States). GSH levels and CD4 and CD8 lymphocyte counts were determined immediately after blood collection.

### Glutathione

Glutathione concentrations were quantified in 200 μl of whole blood using the Ellman reagent 5,5′-ditiobis (2-nitrobenzoic acid) (DTNB). The sulfhydryl group is soluble after placing the sample in a solution of trioxophosphate acid and EDTA. A 500 μl aliquot reacts with 250 μl DTNB to form a yellow chromophore that is read at 412 nm. The concentration of total GSH was calculated by interpolating the absorbance of the samples on a calibration curve with known concentrations of 0, 1, 5, 10, 15, 20, 40, and 50 μmol/L (prepared from a stock solution of 1 mM GSH). GSH levels were corrected according to hemoglobin concentration in each sample, expressed as GSHc.

### Lymphocytes

Lymphocyte subpopulations were detected by flow cytometry using an acoustic focus cytometer (FACS Attune, Applied Biosystems, Foster City, CA, United States). Monoclonal antibodies (Becton Dickinson Biosciences, Franklin Lakes, NJ, United States) that were coupled to different fluorophores were used for each cell subset (anti-CD4-PE and anti-CD8-Alexa488). In 200 μl of whole blood, erythrocytes were lysed with ammonium-chloride-potassium solution, and then leukocytes were resuspended in phosphate saline solution. A 5 μl aliquot of CD4 antibody and 1.25 μl aliquot of CD8 antibody were added in a dark room and incubated at 4°C for 60 min. The samples were fixed with 1% paraformaldehyde and 0.02% sodium azide. A total of 50,000 events were evaluated per sample. Distribution graphs of cell populations were analyzed using (FlowJo 10 software, Ashlan, OR, United States). The results are expressed as a percentage of CD4 and CD8 lymphocytes and the CD4/CD8 ratio.

### Statistical Analysis

The data were analyzed using (STATA 14 software, College Station, TX, United States) (Stata Corp, College Station, TX, United States). The frequency and percentage of categorical variables are presented. Continuous data are presented as means and standard deviations and SEM. The Spearman test was used to analyze correlations. Contingency tables for exact Fischer and χ^2^ tests were created to compare the distribution of categorical variables between groups. Data that followed a normal distribution were analyzed using parametric analysis of variance (ANOVA) and Student’s *t*-test as appropriate. Non-parametric data were analyzed using the Kruskal-Wallis test and Mann-Whitney *U* test. Significant effects in the Kruskal-Wallis test were followed by the Dunn *post hoc* test. Values of *p* ≤ 0.05 were considered statistically significant.

## Results

### General Characteristics

A total of 177 students, 18–23 years of age, participated in the study. The majority of students were women and single, and 35.6% engaged in regular physical activity. A total of 56.5% of the students reported that they lived with their parents, 36.7% lived in a rented room, and 6.7% lived with uncles or grandparents. A fifth of the population was also employed. Substance use was reported by 26.1% of the students, with a higher frequency in men (15.3%) than in women (10.8%; χ^2^ = 4.02, *df* = 1, *p* = 0.04). The substance with the highest frequency of consumption was alcohol, followed by tobacco and marijuana (Table 1). In 20.3% of the students, substances were consumed for pleasure or social use and to a lesser extent because of nervousness (3.5%) or sadness (2.3%).

**TABLE 1:** General features and life activities of the participants (*n* = 177).

Variable	*n*	%
**Gender**		
Women	95	53.7
Men	82	46.3
**Dating relationship**		
No	118	66.7
Yes	59	33.3
**Work**		
No	140	79.1
Yes	37	20.9
**Reason for working**		
School expenses	12	6.8
Self-support	9	5.1
Family expenses	8	4.5
Personal expenses	8	4.5
**Physical activity**		
Never	91	51.4
Regular	63	35.6
Occasional	23	12.9
**Substance use**		
None	131	73.8
Alcohol	22	12.5
Alcohol and tobacco	14	7.9
Tobacco	7	3.9
Marijuana	3	1.8

The results of the psychometric scales showed that perceived stress was present in 77.4% of the students. Anxiety symptoms were present in 71.1% of the students, and depression symptoms were present in 16.4% of the students. The intensity of these symptoms was generally mild. The frequency of students with high self-esteem was higher than the frequency with normal or low self-esteem. Students scored higher on socio-familiar support than social or familiar support separately (Table 2).

**TABLE 2:** Psychometrics of the participants (*n* = 177).

Variable	*n*	%	Mean ± SD
PSS score			15.1 ± 6.0
No stress	40	22.6	7.7 ± 2.4
Mild stress	105	59.3	15.1 ± 2.8
High stress	32	18.1	24.6 ± 3.2
Hamilton score			10.5 ± 6.6
No anxiety	51	28.8	3.6 ± 1.7
Mild anxiety	67	37.8	8.9 ± 1.7
Moderate anxiety	59	33.3	18.1 ± 4.7
Beck score			6.3 ± 6.2
No depression	148	83.6	4.1 ± 2.9
Mild depression	22	12.4	14.2 ± 2.8
Moderate depression	7	3.9	27.4 ± 6.6
Rosenberg score			32.7 ± 5.1
Low self-esteem	14	8.0	23.0 ± 3.3
Normal self-esteem	46	25.9	28.0 ± 1.5
High self-esteem	117	66.1	35.7 ± 2.9
FSFSS score			
Social support	–	–	26.8 ± 6.1
Familiar support	–	–	31.4 ± 6.9
Socio-familiar support	–	–	58.3 ± 11.2

**FIGURE 1:**
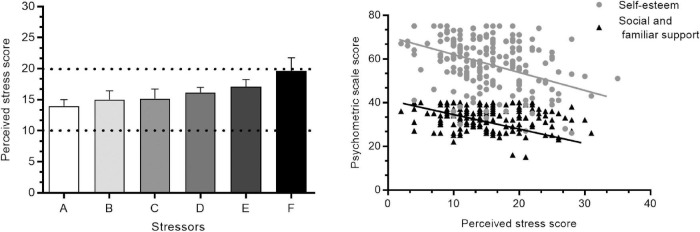
Perceived stress **(left)** and psychometric correlation **(right)**. (A) Exams. (B) Eating schedule. (C) Academic failure. (D) Workload. (E) Time distribution. (F) Acceptance by peers. Dash lines show the interval of mild stress score. The results are expressed as mean ± SEM.

The students reported different stressors, such as the number of homework assignments (29.8%), exams (19.8%), the time to complete school work (19.2%), the short time to eat (14.5%), a fear of failure (11.3%), and peer acceptance (5.3%). Although PSS-10 scores reflected mild stress, no differences among these stressors were observed (*F*_5,145_ = 1.56, *p* = 0.17; Figure 1).

Perceived Stress Scale −10 scores were significantly higher in women than in men (*t*_175_ = 2.59, *p* = 0.01). No sex differences in anxiety (*U*_175_ = 0.37, *p* = 0.70) or depression (*U*_175_ = 0.96, *p* = 0.33) symptom scores were found. In women, PSS-10 scores were similar among the four phases of the menstrual cycle (*F*_3,91_ = 2.2, *p* = 0.08). Lower Hamilton Anxiety Scale scores (*H*_3_ = 10.25, *p* = 0.01) and lower BDI-I scores (*H*_3_ = 8.36, *p* = 0.03) were observed in the follicular phase compared with the premenstrual, menstrual, and luteal phases. Neither physical activity nor substance use was associated with emotional symptoms. Figure 1 shows that stress was negatively correlated with self-esteem (Spearman’s rho = −0.65, *p* < 0.0001) and socio-familiar support (Spearman’s rho = −0.29, *p* < 0.0001).

### Group Comparisons

**FIGURE 2:**
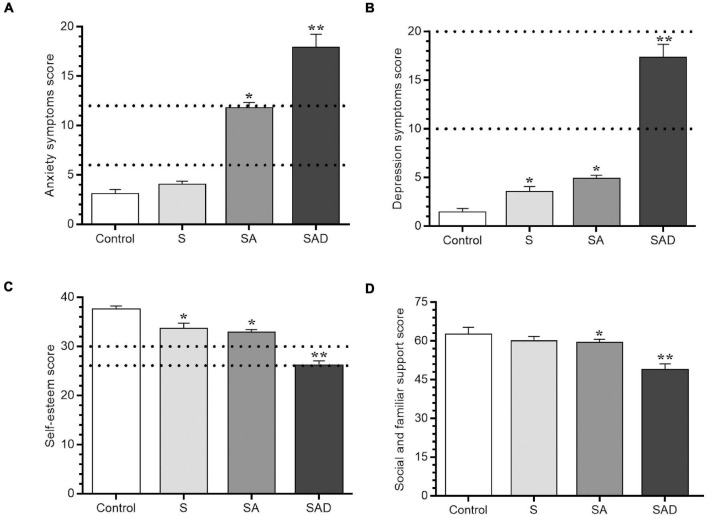
Socio-affective scores for **(A)** anxiety, **(B)** depression, **(C)** self-esteem, and **(D)** social and familiar support. The following groups were compared: S, stress; SA, stress and anxiety symptoms; SAD, stress, anxiety, and depression symptoms. Dashed lines show the interval of a mild level of anxiety and depression symptoms and the average self-esteem score. *p < 0.05 vs. S and control groups; **p < 0.001 vs. SA, S, and control groups (Dunn test). The results are expressed as mean ± SEM.

Based on severity, among the total number of students in this study, 14.1% were assigned to the control group (*n* = 25), 14.7% were assigned to the S group (*n* = 26), 54.8% were assigned to the SA group (*n* = 97), and 16.4% were assigned to the SAD group (*n* = 29). The distribution of men and women was similar between groups (χ^2^ = 3.36, *df* = 3, *p* = 0.33). Significantly higher anxiety (*H*_3_ = 117.57, *p* = 0.0001) and depression (*H*_3_ = 93.27, *p* = 0.0001) symptoms were observed in the SAD group compared with the control and SA groups (Figures 2A,B).

The analysis of item 9 of the BDI-I showed that 11.3% of the participants (*n* = 20) expressed suicidal ideation. Among these participants, 7.9% were in the SAD group, and 3.4% were in the SA and S groups. Students in the S, SA, and SAD groups had lower self-esteem than students in the control group (*H*_3_ = 66.6, *p* = 0.0001; Figure 2C). Students in the SAD group reported less socio-familiar support (*H*_3_ = 23.44, *p* = 0.0001; Figure 2D).

Hemoglobin, GSH, and GSHc levels, leukocyte, total lymphocyte, and CD4 and CD8 lymphocyte counts, and the CD4/CD8 ratio are shown in Table 3. All of these parameters were within the reference range and within the average value for healthy subjects.

**TABLE 3:** Hematological and biochemical parameters (*n* = 177).

	Mean ± SD	Reference range
**Hemoglobin (g/dl)**		
Women	13.6 ± 0.8	12–16^a^
Men	15.9 ± 0.7	14–17^a^
Total GSH (μmol/L)	7.8 ± 6.3	8.19 ± 1.4^b,^ ^†^
GSHc (μmol/gHb)	5.2 ± 4.2	7.67 ± 0.032^c,^ ^†^
Leukocytes (cells^3^/ml)	6.3 ± 1.3	5–10^a^
Lymphocytes (%)	27.8 ± 10.1	18–54^d^
% CD4	29.5 ± 9.6	23–62^d^
% CD8	14.2 ± 4.9	14–44^d^
CD4/CD8 ratio	2.1 ± 0.4	0.6–4.4*^d^*

*^†^Average value in healthy individuals (mean ± SD).*

*^a^National Cancer Institute, SEER Training Modules (2021).*

*^b^Richie et al. (1996).*

*^c^Carru et al. (2002).*

*^d^Valiathan et al. (2014).*

**FIGURE 3:**
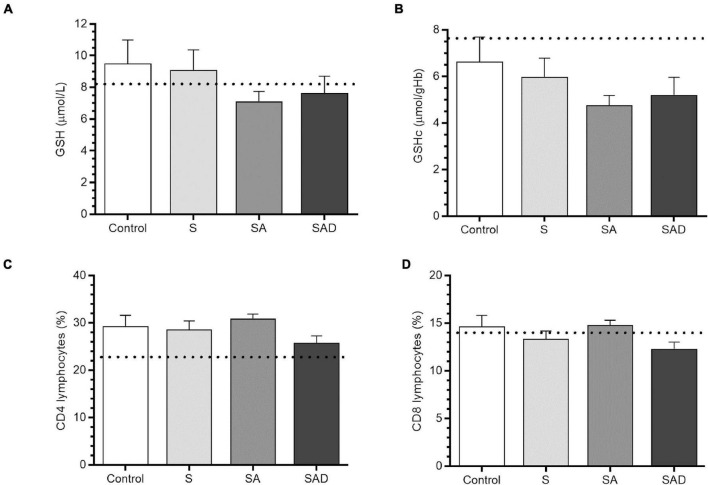
Antioxidant **(A,B)** and immune **(C,D)** parameters. The following groups were compared: S, stress; SA, stress and anxiety symptoms; SAD, stress, anxiety, and depression symptoms. The dashed line shows the average value for healthy subjects **(A,B)** and the lower limit of the reference range **(C,D)**. The results are expressed as mean ± SEM.

The statistical analysis did not show differences in GSH (*H*_3_ = 6.02, *p* = 0.11) or GSHc (*H*_3_ = 5.98, *p* = 0.11) levels among groups (Figures 3A,B). A trend toward a decrease in GSHc levels was found in the SA and SAD groups compared with the control and S groups (*H*_2_ = 5.40, *p* = 0.06). GSHc levels were not influenced by substance use (*U*_175_ = 0.197, *p* = 0.84) or physical activity (*U*_175_ = 0.99, *p* = 0.31). GSHc levels were not different between men and women (*U*_175_ = −0.97, *p* = 0.33). GSHc levels did not differ between menstrual cycle phases (*H*_3_ = 2.240, *p* = 0.52).

Trends toward decreases in the percentage of CD4 (*H*_3_ = 7.15, *p* = 0.06) and CD8 (*H*_3_ = 6.30, *p* = 0.09) were found in the SAD group compared with the control and SA groups (Figures 3C,D). The CD4/CD8 ratio was not significantly different between groups (*H*_3_ = 0.858, *p* = 0.83).

Correlations between psychometric scores, GSH levels, and lymphocyte counts were not significant. A trend was observed between GSH levels and anxiety symptom scores (Spearman’s rho = −0.14, *p* = 0.0529) and between GSHc levels and anxiety symptom scores (Spearman’s rho = −0.14, *p* = 0.0559).

## Discussion

This study evaluated associations between GSH concentrations, CD4 and CD8 lymphocyte counts, self-esteem, socio-familiar support, and symptoms of anxiety and depression in young adults who apparently had no current clinical diagnosis. The youth stage of development is a transition period that is characterized by a series of biological, social, familiar, and economic events that can affect mental health. The present results showed that a high percentage (77.4%) of students presented mild-intensity stress. This percentage was higher than in previous reports in the Mexican population, in which 65% of young people presented stress that derived from school activities (Halgravez Perea et al., 2016; Luna et al., 2020). In other Latin American countries, the frequency of stress among students was reported to be 38.8–85% (Jerez-Mendoza and Oyarzo-Barría, 2015; Peltzer and Pengpid, 2015). In Caucasian and Egyptian students, the prevalence was reported to be 41and 60%, respectively (Abdel Wahed and Hassan, 2017; Fawzy and Hamed, 2017). Although this sample was relatively small, our findings were similarly high for college students in other countries.

In this study, the students did not exhibit a significant increase in perceived stress according to the source of stress, but the perceived need for acceptance by peers was a notable stressor in this study. This aspect reflects social belonging and the process of adapting to a new school environment (De la Fuente et al., 2014). When there is a minimal capacity of self-control at the psychic level in stressful situations, somatic manifestations can occur that interfere with academic performance and the establishment of social relationships and predict the development of emotional and affective disorders in the long term (Bohman et al., 2018; León Hernández et al., 2019).

The 71.1% of the students who presented mild to moderate symptoms of anxiety was higher than in previous studies with similar college populations, in which the frequencies ranged from 19 to 41% (Rosas-Santiago et al., 2016; Tijerina González et al., 2018), and higher than in Liu et al. (2019), who reported that 40% of Chinese students exhibited moderate to severe anxiety symptoms. Anxiety symptoms were reported to be associated with academic issues in approximately 8% of French undergraduate students in sciences, humanities, medicine, political science, sports science, engineering, and business (Tran et al., 2017). The prevalence was notably higher (63%) among medical students of Saudi and non-Saudi nationalities (Kulsoom and Afsar, 2015) and notably higher (72%) among Pakistani medical students (Azim and Baig, 2019), which was similar to the present findings.

Importantly, 16.3% of the students in this study with mild to moderate depression symptoms reported stress and also symptoms of anxiety. This percentage was similar to the 16% prevalence of depression symptoms that was reported in students from the beginning to end of college (Romo-Nava et al., 2019), the severity of which was mild to moderate (Rosas-Santiago et al., 2016). These percentages are higher than the 4.2% prevalence in an Asian young-adult population (18–25 years of age) who presented anxiety and depression (Risal et al., 2016). In addition to anxiety symptoms, chronic stress increased the development of depression symptoms by 35–50% (Ngasa et al., 2017). Because of the cross-sectional design of this study, however, we cannot establish when and which symptoms occurred first in the participants. Anxiety symptoms may have preceded depressive status because no isolated depression symptoms were recorded in this study. Therefore, as a consequence of anxiety, a reduction of cognitive function could have occurred, similar to a previous study of prospective memory in undergraduate students (Bowman et al., 2019).

The prevalence of anxiety or depression is higher in women than in men (Romo-Nava et al., 2019; Ramón-Arbués et al., 2020). However, we considered that the lack of a significant difference in symptoms between sexes may be attributable to the mild to moderate severity of symptoms, which is different from the clinical population, who usually suffers from a higher severity of these symptoms. Additionally, participants are exposed to similar demands in the school environment, and personal or familiar support likely mediates the emotional response to stress.

In this study, socio-familiar support was high in the control group and negatively correlated with PSS-10 scores. Thus, an adequate level of socio-familiar support, combined with an optimal level of trust, is a mechanism of emotion regulation and a significant predictor of emotional health in students. This finding is similar to studies of American, Lithuanian, and Korean students (d’Arbeloff et al., 2018; Novak et al., 2018; Choi et al., 2019). Moreover, high socio-familiar support positively correlates with motivation and better performance (Almalki, 2019).

Low motivation is related to health-risk behaviors, such as addiction, alcohol consumption, and poor eating habits (Aoun et al., 2019; Begdache et al., 2019). In this study, alcohol and tobacco consumption was reported in 26.1% of the students who had symptoms of depression and anxiety. Although causal factors are difficult to establish, alcohol and tobacco consumption has been shown to increase in situations that generate anxiety or alter emotional states (Kulsoom and Afsar, 2015; Santangelo et al., 2018). El Ansari et al. (2013) reported alcohol consumption (i.e., low episodic drinking) in 40% of students during the first years of university as a result of doubts about knowledge and skills (i.e., low self-esteem) and a lack of professional identity, but alcohol consumption decreased as school progressed. Therefore, we expect the low frequency of students consuming alcohol and tobacco may not increase if these students address strategies for coping with stress.

The control group in this study suggested that an optimum level of self-esteem that is higher than the normal range is an important factor to reduce unhealthy practices and psychiatric problems. Self-esteem fully mediated the development of depression symptoms, which is consistent with previous studies of students from Korea and the Republic of Cyprus (Choi et al., 2019; Ioannou et al., 2019). Although self-esteem scores in the SAD group were within the normal range, they may have mitigated the worsening of symptomatology because self-esteem acts as a mediating element in stressful situations.

Studies have investigated the influence of school, social, and family environments on emotional responses to stress in students. Stress can also manifest physiologically. We evaluated antioxidant concentrations and immune system parameters, to associate them with the emotional and affective states of the students. During the stress response, the loss of negative feedback of the HPA axis intensifies glucocorticoid secretion, increases the production of reactive oxygen species (Coronado et al., 2015), and produces oxidative stress when antioxidants decrease, including GSH (Godlewska et al., 2015). GSH and GSHc concentrations that were quantified in this study did not significantly differ between groups, but they were the lowest in groups who perceived stress and presented symptoms of anxiety and depression. This indicates that the antioxidant GSH is slightly different according to perceptions of stress and symptoms of anxiety and depression, but it is not a marker when the severity of symptoms is mild to moderate. At the time of this study, the population of students did not have chronic or acute illnesses that could modify GSH levels. Thus, we propose that GSH concentrations are closely related to affective and emotional symptoms in students. We predict that GSH levels could be lower in later stages such as anxiety and depression progression. If proper interventions are not implemented to address symptoms of the development of anxiety and depression, then these symptoms could become more severe, and physiological changes may be evident, including lower levels of GSH, that are comparable with the adult population that is clinically diagnosed. Oxidative stress markers are clearly associated with affective disorders rather than antioxidants. For example, F2-isoprostane and oxidized GSH have been positively associated with anxiety scores but a lack of differences in plasma GSH levels was reported in major depressive disorder patients with anxiety symptoms (Steenkamp et al., 2017). Other studies that assessed cortical GSH levels by proton magnetic resonance spectroscopy suggested that GSH may be a biomarker of depression, with lower levels in patients with major depression (Godlewska et al., 2015; Freed et al., 2017), which is consistent with its negative correlation with the severity of symptoms (Lapidus et al., 2014). Findings in peripheral blood samples may not be sufficiently robust to extrapolate to brain physiology.

Other biological processes beyond oxidative stress and antioxidant function are related to mental disorders, such as inflammation, which can reduce the biosynthesis and availability of neurotransmitters and affect neuroplasticity (Sperner-Unterweger et al., 2014; Marx et al., 2017). Markers of immune system function reveal alterations during the stress response, which have also been related to psychopathological disorders (Leonard and Maes, 2012; Fagniart et al., 2016). Patients with depression have lower CD4 or CD8 lymphocyte counts, which are associated with other markers of immune system function, such as interleukins and proinflammatory cytokines (Grosse et al., 2016). Decreases in CD4 and CD8 lymphocytes have been associated with the severity of depressive symptomatology. By contrast, antidepressant pharmacotherapy improves depressive symptomatology and increases CD8 lymphocytes 4–8 weeks post-treatment (Goyal et al., 2017; Jeon et al., 2018). In this study, no sex differences in the percentages of lymphocyte populations were found between groups of non-diagnosed students. In patients who were clinically diagnosed with depression, significant differences in lymphocytes were found between male and female patients (Fagniart et al., 2016). Less information is available about sex differences in immune parameters. In this study, lymphocyte counts were the lowest in the SAD group compared with the other groups, albeit not significantly. This trend toward an association between low lymphocyte counts and depression symptoms in college students may become significant if the symptoms of the students progress to a clinical diagnosis that is associated with an immunosuppressive state. Leukocyte fractions, such as lymphocytes, were positively associated with mental wellbeing in a general population (Gialluisi et al., 2020). Other peripherical blood parameters support the hypothesis that psychological factors influence subclinical low-grade inflammation and immune function, and depression can or cannot course with immune alterations. The relationship between mental health and inflammation has been suggested to be bidirectional, in which both psychological and inflammatory conditions can amplify their effects. Additionally, among all cases of depression, approximately 40% have high immune cell counts, whereas the other 60% do not, indicating that certain subpopulations have different abnormal immune cell counts (Lynall et al., 2020). Lifestyle may be a therapeutic target for interventions to prevent mental health disorders and chronic health conditions (Gialluisi et al., 2020).

This study has limitations. First, this study lacked a clinical screening or structured interview to corroborate the answers of the participants about a psychiatric or affective diagnosis. These findings suggest that most of the participants did not have an anxiety or depression disorder. Although the anxiety and depression symptom instruments that were used have been validated, they are self-report questionnaires and only evaluated symptom severity, and they do not necessarily have diagnostic value. Thus, the participants in this study self-reported that they do not have a psychiatric diagnosis, but some of them could be underdiagnosed. Future studies should incorporate clinical interviews to conduct comparisons with healthy students. Second, this study lacked a clinical evaluation to discard possible chronic diseases. The students reported the absence of any medical condition. Normal ranges of immune and hematological parameters are well accepted as basic screenings of a good immune system and health status. Future studies should conduct a thorough medical evaluation even when the target population is considered healthy. Third, there are few reports on serum GSH measurements in non-clinical individuals to make comparisons with our findings. GSH levels in the central nervous system that are assessed in psychiatric patients by spectroscopy techniques are not equivalent to serum concentrations. Nonetheless, the interpretation of central and peripheral GSH levels may be presumed to be in the same direction with regard to affective symptoms. Fourth, although the sample size achieved the necessary statistical power to support the results, future studies should include larger sample sizes to analyze cohorts and corroborate the directions of the present findings.

Despite the above limitations, we found that a combination of psychiatric, cognitive, and social factors plays an important role in the mental health of college students, which is consistent with previous reports (Ramón-Arbués et al., 2020). Together with physiological and biochemical evaluations, our psychometric findings could predict negative outcomes that affect the performance and quality of life of an individual. Although the GSH, CD4, and CD8 findings did not reach statistical significance, they could have predictive value for a population that is not yet clinically diagnosed or not yet treated and who could present an increase in symptom severity and progress to a clinically defined psychiatric disorder. Such symptoms could be managed by psychological therapy to effectively restore immune system alterations in young adults (Gialluisi et al., 2020; Pearlstein et al., 2020). The present findings highlight the importance of future strategies to preserve mental health. The impact of oxidative stress and immune function on the central nervous system and their association with depression and anxiety are well known (Haapakoski et al., 2016; Lindqvist et al., 2017; van Velzen et al., 2017; Jeon et al., 2018; Mazza et al., 2020). We suggest screening early symptoms of psychopathology in combination with immune and antioxidant parameters to better understand the complex inflammation theory of depression.

## Conclusion

The relationship between antioxidant/immune parameters and socio-affective scores was latent in undiagnosed college students who might later develop affective disorders. Our findings on emotional health in college students could suggest incipient stages of affective disorders for which stress management strategies could be incorporated to cope with the stress of academic load and monitor negative changes in physiological state.

## Data Availability Statement

The raw data supporting the conclusions of this article will be made available by the authors, without undue reservation.

## Ethics Statement

The studies involving human participants were reviewed and approved by the Research Ethics Committee of the Institute of Health Sciences of the Universidad Veracruzana (registration CE-ICS No._002, 2018). The patients/participants provided their written informed consent to participate in this study.

## Author Contributions

CB-V, BB-M, and OV performed conceptualization, methodology, formal analysis, writing the original draft, and funding acquisition. OV performed validation. CB-V, JV-H, and MX-G performed investigation and supervision. EG-M, JR-L, EH-H, and LV supported in finding resources and writing—review and editing. All authors fully reviewed and approved the submitted manuscript.

## Conflict of Interest

The authors declare that the research was conducted in the absence of any commercial or financial relationships that could be construed as a potential conflict of interest.

## Publisher’s Note

All claims expressed in this article are solely those of the authors and do not necessarily represent those of their affiliated organizations, or those of the publisher, the editors and the reviewers. Any product that may be evaluated in this article, or claim that may be made by its manufacturer, is not guaranteed or endorsed by the publisher.
